# A Hybrid Method for Performance Degradation Probability Prediction of Proton Exchange Membrane Fuel Cell

**DOI:** 10.3390/membranes13040426

**Published:** 2023-04-12

**Authors:** Yanyan Hu, Li Zhang, Yunpeng Jiang, Kaixiang Peng, Zengwang Jin

**Affiliations:** 1 School of Intelligence Science and Technology, University of Science and Technology Beijing, Beijing 100083, China; 2 Institute of Artificial Intelligence, University of Science and Technology Beijing, Beijing 100083, China; 3 SPIC Digital Technology Co., Ltd., Beijing 100080, China; 4 School of Automation and Electrical Engineering, University of Science and Technology Beijing, Beijing 100083, China; 5 School of Cybersecurity, Northwestern Polytechnical University, Xi’an 710072, China; 6 National Engineering Laboratory for Integrated Aero-Space-Ground-Ocean Big Data Application Technology, Northwestern Polytechnical University, Xi’an 710072, China

**Keywords:** degradation prediction, proton exchange membrane fuel cell, Wiener process, transformer, Monte Carlo dropout

## Abstract

The proton exchange membrane fuel cell (PEMFC) is a promising power source, but the short lifespan and high maintenance cost restrict its development and widespread application. Performance degradation prediction is an effective technique to extend the lifespan and reduce the maintenance cost of PEMFC. This paper proposed a novel hybrid method for the performance degradation prediction of PEMFC. Firstly, considering the randomness of PEMFC degradation, a Wiener process model is established to describe the degradation of the aging factor. Secondly, the unscented Kalman filter algorithm is used to estimate the degradation state of the aging factor from monitoring voltage. Then, in order to predict the degradation state of PEMFC, the transformer structure is used to capture the data characteristics and fluctuations of the aging factor. To quantify the uncertainty of the predicted results, we also add the Monte Carlo dropout technology to the transformer to obtain the confidence interval of the predicted result. Finally, the effectiveness and superiority of the proposed method are verified on the experimental datasets.

## 1. Introduction

The proton exchange membrane fuel cell (PEMFC) has many excellent properties: renewable, no pollution, high energy conversion rate, fast start-up speed, and low operating temperature [1,2]. It is regarded as a promising power conversion device, widely used in the military, vehicle transportation, portable power supply and many other fields [3,4,5]. Nevertheless, the short lifespan is one of the key barriers to its large-scale commercialization [6]. Performance degradation prediction has the ability to predict the future performance degradation trend and the failure time of PEMFC. Based on the prediction result of performance degradation, appropriate maintenance measures could be taken in advance to extend the lifetime. Consequently, performance degradation prediction is an effective way to guarantee the safe and reliable operation of PEMFC, and also directly promotes its commercial application.

The performance degradation prediction of PEMFC has yielded many fruitful results. The existing PEMFC prediction methods can be generally divided into three categories: model-based methods, data-driven methods and model and data hybrid-driven methods.

The model-based methods considered from the inside of the system usually establish mechanism models or empirical models according to the degradation mechanisms of the equipment or the degradation trends of the monitoring data, and describe the changes in the inside of the systems with specific mathematical expressions in order to predict the failure time. Such mechanism models can complete the degradation prediction task without a large amount of online data, such as the method based on particle filter (PF) in [7]. However, due to the complexity of the internal structures of the systems, it is often difficult to establish such mechanism models of the systems. Later, scholars generally improved physical models as empirical models, where model parameters were processed through fuzzy or estimated online based on monitoring data. In [8], the unscented Kalman filter (UKF) algorithm was combined with three empirical models, including linear, logarithmic and exponential models, to approximate the aging process and thus predict the performance degradation trend. Model-based methods have the advantage of requiring less data and being able to generate analytical predictions for remaining useful life (RUL). Nevertheless, the majority of machines undergo a gradual degradation process leading up to failure, which can pose a challenge for model-based methods to accurately predict due to the intricate degradation mechanisms involved. In addition, these methods can only obtain the general trend of PEMFC degradation and cannot capture the local characteristics of degradation information well.

Different from model-based methods, data-driven methods do not need an in-depth analysis of the mechanism and law of equipment degradation but can directly predict the degradation trend of PEMFC by machine learning methods based on monitoring data, such as relevance vector machine (RVM) [9,10], echo state network (ESN) [11,12], extreme learning machine (ELM) [13,14], etc. In recent years, with the continuous development of deep learning theory, neural network models have been gradually introduced into the prediction of the degradation trend of equipment, for example, convolutional neural network (CNN) [15], long- and short-term memory (LSTM) [16,17,18], gated recurrent unit (GRU) [19], etc. With the continuous enhancement of the data mining ability of deep learning models, these kinds of methods gradually give up the dependence on expert knowledge, and can directly extract health indicators from the original data as the input of the neural network to learn the degradation law of the equipment and realize the degradation prediction. Although these methods can use the abundant monitoring data obtained by sensors and quickly mine the degradation information of the equipment health status, it can only obtain the point prediction result, and the uncertainty in the prediction process is often ignored.

To deal with the aforementioned constraints and integrate the strengths of both model-based and data-driven prediction methods, some scholars have proposed hybrid prediction approaches. Cheng et al. [20] proposed a hybrid prediction method combining least square support vector (LSSVM) and regularized particle filter (RPF). Liu et al. [21] used adaptive neuro-fuzzy inference system (ANFIS) to predict the long-term degradation trend of stack voltage, and then estimated the aging state of PEMFC based on the predicted results using an adaptive unscented Kalman filter (AUKF) algorithm, ultimately determining the RUL. Xie et al. [22] proposed a fusion prognostic method based on PF and LSTM; PF and LSTM independently realize the prediction of RUL, and then fuse under the given fusion principle to obtain more accurate prediction results. Ma et al. [23] integrated the extended Kalman filter (EKF) with LSTM algorithms to improve the accuracy of prediction result. EKF was used to estimate the system state to further obtain the estimated voltage, while the voltage result predicted by LSTM was used as the new observations for EKF to continue to estimate the system state. In [24], a decomposition prediction framework was proposed with consideration of the voltage recovery phenomenon. The EKF algorithm was used to predict the overall trend of voltage degradation, and LSTM was used to predict the local recovery information of voltage. Most of the existing prediction methods usually adopt the LSTM to learn the degradation trend of PEMFC. As a particular kind of recurrent neural network (RNN), LSTM has obvious superiority in time series processing and is therefore widely used in the field of degradation prediction of PEMFC. When processing time series problems, the training of LSTM is iterative. The output of LSTM at the next moment depends on the output of the previous moment. This mode operates in a sequential manner, which will significantly impede the computational efficiency of the model. Additionally, LSTM relies on recurrent connections; it is prone to the issue of gradient vanishing, which causes the network to forget important information and become less effective at capturing long-term dependencies. The  ability of the model to retain and utilize previous information may deteriorate over time, leading to suboptimal performance on lengthy input sequences.

Transformer [25] is a recently proposed network based on self-attention mechanism. This model can not only deal with the change of long-term dependence relationship over time effectively but also improve the operation efficiency through parallel computing. Compared to RNN, transformer breaks the limitation that RNN models cannot be computed in parallel. Compared with CNN, transformer can capture remote features since its field of view is not limited by the size of convolution kernel when processing temporal features. The transformer network has been widely used in computer vision (CV) and natural language processing (NLP), but it is rarely used in PEMFC degradation prediction. In view of the above problem, this paper proposes the prediction model based on transformer as the data-driven method to predict the degradation trend of PEMFC. In addition, the attention mechanism of transformer makes the neural network more focused on important time steps and has better characteristics in global information capture and long-term dependence.

With regard to the performance degradation prediction problem, as we all known that the aging process of the PEMFC is affected by many uncertain factors due to the comprehensive effect of internal structure and external environment, such as the temporal variability, measurement variability, and unit-to-unit variability. So it is more reasonable to use a stochastic process model to model the degradation of PEMFC. The Wiener process can model the non-monotone degradation process and has a clear physical explanation, which is widely used in bearing, turbofan engine and other fields. Therefore, in order to describe the uncertainty of the whole degradation process, a Wiener process model is built in this paper.

Another problem with performance degradation prediction is that although data-driven methods can accurately predict the degradation trend, the uncertainty quantification and reliability of degradation prediction are mostly ignored, and only the point prediction result can be obtained. Point prediction is only a deterministic value, which is difficult to provide sufficient guidance for equipment maintenance in practice. At present, the methods of quantitative uncertainty prediction mainly include Bayesian estimation, Gaussian process and hidden Markov method. The Bayesian neural network introduces the probability distribution to network parameters and trains the model by approximating the posterior distribution based on variational inference technique. Although this method can obtain the uncertainty of the network model, it needs to optimize the mean and standard error of the probability distribution at the same time. Many extra parameters are introduced that increase the training time [26]. Applying dropout to neural networks is equivalent to Bayesian variational inference, and an approximate uncertainty representation similar to Bayesian variational inference can be obtained by sampling techniques [27]. Therefore, the uncertainty of prediction result is quantified using the Monte Carlo dropout (MC-dropout) technique in this paper.

According to the above descriptions, a hybrid driven method for performance degradation prediction of PEMFC is proposed in this paper. Firstly, a linear Wiener process considering multiple variability sources is constructed to adaptively estimate the degradation states based on the UKF algorithm and expectation maximization (EM) algorithm. Secondly, the transformer network is used to learn the degradation characteristics and predict the long-term degradation trend of PEMFC. The MC-dropout technology is added to the network to quantify the uncertainty of the predicted results and obtain a 95% confidence interval. Finally, the proposed method is verified on experimental datasets under both static and dynamic conditions and compared with the classic LSTM method. Experimental results show that the proposed method can achieve satisfactory prediction performance under both scenarios and has fewer prediction errors compared with LSTM, which illustrates the effectiveness and superiority of the proposed method. The main contributions of this paper are summarized as follows:

(1) With consideration of the uncertainties in the degradation process, a Wiener process model is established to describe the overall degradation trend of PEMFC and multiple kinds of variability sources are adequately considered in the model.
(2) To overcome the disadvantage of LSTM in parallel processing, a degradation prediction model is established by transformer, which is used to predict the degradation trend and capture the local fluctuation information.
(3) The MC-dropout is added in transformer network to quantify the uncertainty of the prediction results in order to provide more effective decision support for practical engineering applications.

The organization of this paper is as follows. The Wiener process model is established in Section 2. In Section 3, the degradation state estimation and model parameter estimation methods are introduced. Section 4 introduces the transformer network with MC-dropout technology. Section 5 provides the experimental results to demonstrate the proposed method. Section 6 concludes this paper.

## 2. Degradation Modeling of PEMFC

The voltage of PEMFC is one of the quantities that can be easily measured during its long-term operation and a semi-empirical voltage model [28] of PEMFC can be expressed as

(1) $$V_{st}=n_{cell}\left(E_{0}-iR-AT\ln\left(\frac{i}{i_{0}}\right)-BT\ln\left(1-\frac{i}{i_{L}}\right)\right)$$

where $V_{st}$ is the output stack voltage, $n_{cell}$ is the cell number, *A* is the Tafel constant and *B* is the concentration constant, respectively. The stack current *i* and operating temperature *T* can be measured by sensors during the operation of PEMFC. The open circuit voltage $E_{0}$ and the exchange current $i_{0}$ can be identified by genetic algorithm [29] and Levenberg–Marquardt method [30].

It is demonstrated in [30,31] that the resistance *R* and the current density $i_{L}$ are the only two parameters which have significant changes during the degradation of PEMFC, no matter whether the load is constant or dynamic. $E_{0}$ and $i_{0}$ can be considered constant without obvious changes. In addition, studies have found that the variations of *R* and $i_{L}$ are quite similar and highly correlated with each other [28]. In this paper, a single variable α is adopted to describe the change trend of aging parameters *R* and $i_{L}$:

(2) $$R\left(t\right)=R_{0}\left(1+\alpha \left(t\right)\right)$$

(3) $$i_{L}\left(t\right)=i_{L_{0}}\left(1-\alpha \left(t\right)\right)$$

where $R_{0}$ and $i_{L_{0}}$ are initial values. α represents the change of aging parameters, which can reflect the inner degradation state of PEMFC. The larger the value of α, the more severe the degradation of PEMFC.

Considering that the degradation of PEMFC is stochastic, we use a Wiener process to model the degradation trend of aging factor α:

(4) α(t)=α(0)+θt+σB(t)

where θ is the drift coefficient denoting the degradation rate of α, σ>0 is the diffusion coefficient. Standard Brownian motion {B(t),t>0} describes the stochastic dynamics of degradation process and the temporal variability of degradation process with time. α(0) is the initial degradation state. Here, θ is taken as a stochastic variable to describe the unit-to-unit variability of the degradation process with $\theta ∼N(\mu_{\theta },{\delta_{\theta }}^{2})$, where $\mu_{\theta }$ is the averaged degradation rate with ${\delta_{\theta }}^{2}$ as its variance. In order to describe the change of drift coefficient over time, its updating mechanism over time is established as follows:

(5) $$\theta \left(t\right)=\theta \left(t-1\right)+v_{t}$$

where $v_{t}∼N\left(0,v^{2}\right)$.

## 3. State of Health Estimation and Parameter Estimation

Based on the above description, the aging state estimation of PEMFC can be expressed as a non-linear system:

(6) $$\left\{\begin{array}{l} X_{k}=AX_{k-1}+W_{k}\\ V_{k}=h\left(X_{k}\right)+\Gamma_{k} \end{array}\right.$$

$$X_{k}=\left[\begin{array}{c} \alpha_{k}\\ \theta_{k} \end{array}\right],A=\left[\begin{array}{cc} 1 & t_{k}-t_{k-1}\\ 0 & 1 \end{array}\right],$$

$$W_{k}=\left[\begin{array}{c} \sigma [B\left(t_{k}\right)-B\left(t_{k-1}\right)]\\ v_{k} \end{array}\right],\Phi_{k}=\left[\begin{array}{cc} \sigma^{2}\left(t_{k}-t_{k-1}\right) & 0\\ 0 & \upsilon^{2} \end{array}\right].$$

(7) $$h\left(X_{k}\right)=n_{cell}\left(E_{0}-i_{k}R_{0}\left(1+\alpha_{k}\right)-AT\ln\left(\frac{i_{k}}{i_{0}}\right)-BT\ln\left(1-\frac{i_{k}}{i_{L0}\left(1-\alpha_{k}\right)}\right)\right)$$

where $X_{k}$ represents the aging state of PEMFC, $V_{k}$ represents the stack voltage, $W_{k}$ represents the process noise with the variances of $\Phi_{k}$, and $\Gamma_{k}$ represents the measurement noise with the variances of *R* that can reflect the measurement variability.

UKF is a non-linear filtering algorithm [32]. According to the symmetric sampling rule, UKF obtains a set of sigma points with different weights by using unscented transform to approximate the probability density function of the Gaussian distribution state, which reflects the probability density distribution of the transformation state of the system. UKF avoids the process of finding the inverse matrix. Compared with EKF, it improves the estimation accuracy and reduces the calculation time. According to (6), the degradation state of PEMFC is estimated by UKF algorithm in this paper. The procedures of the UKF algorithm are shown in Algorithm  1.
**Algorithm 1** The procedures of the UKF algorithm**1. Initialization (k = 0)**:${\hat{X}}_{0}=E\left(X_{0}\right)$, $P_{0}=E\left[\left(X_{0}-{\hat{X}}_{0}\right){\left(X_{0}-{\hat{X}}_{0}\right)}^{T}\right]$**2. Time update:**${\hat{X}}_{k|k-1}=A{\hat{X}}_{k-1|k-1}$,   $P_{k|k-1}=AP_{k-1|k-1}A^{T}+\Phi$**3. Sigma points and weights calculation:**(1) $\{X_{k|k-1}^{\left(0\right)}={\hat{X}}_{k|k-1},\;i=0$; $X_{k|k-1}^{\left(i\right)}={\hat{X}}_{k|k-1}+(\sqrt{{\left(n+\lambda \right)P)}_{i}},\;i=1\ldots n$; $X_{k|k-1}^{\left(i\right)}={\hat{X}}_{k|k-1}-(\sqrt{{\left(n+\lambda \right)P)}_{i}},\;i=n+1\ldots 2n$where ${\left(P\right)}^{T}\left(P\right)=P$, ${\left(P\right)}_{i}$ is the *i*th column of the square root of the matrix P.(2) $w_{m}^{\left(0\right)}=\lambda /n+\lambda$; $w_{c}^{\left(0\right)}=\lambda /n+\lambda +\left(1-\tau^{2}+\beta \right)$; $w_{m}^{\left(i\right)}=w_{c}^{\left(i\right)}=\lambda /2\left(n+\lambda \right),\;i=1\ldots 2n$,where $\lambda =\tau^{2}\left(n+\kappa \right)$ is the scaling parameter, and the other parameters are generally set to $\tau^{2}=0.01,\;\beta =0,\;\left(n+\lambda \right)P$ = 3**4. Measurement update**$V_{k|k-1}^{\left(i\right)}=h\left[X_{k|k-1}^{\left(i\right)}\right]$${\bar{V}}_{k|k-1}=\sum_{i=0}^{2n}w_{m}^{\left(i\right)}V_{k|k-1}^{\left(i\right)}$$P_{V_{k}V_{k}}=\sum_{i=0}^{2n}w_{c}^{\left(i\right)}\left[V_{k|k-1}^{\left(i\right)}-{\bar{V}}_{k|k-1}\right]{\left[V_{k|k-1}^{\left(i\right)}-{\bar{V}}_{k|k-1}\right]}^{T}+R$$P_{X_{k}V_{k}}=\sum_{i=0}^{2n}w_{c}^{\left(i\right)}\left[X_{k|k-1}^{\left(i\right)}-{\hat{X}}_{k|k-1}\right]{\left[V_{k|k-1}^{\left(i\right)}-{\bar{V}}_{k|k-1}\right]}^{T}$$K_{k}=P_{X_{k}V_{k}}P_{V_{k}V_{k}}^{-1}$${\hat{X}}_{k|k}={\hat{X}}_{k|k-1}+K_{k}\left[V_{k}-{\bar{V}}_{k}\right]$$P_{k|k}=P_{k|k-1}-P_{V_{k}V_{k}}K_{k}K_{k}^{T}$

The degradation model is initialized after model parameter $\mu_{\theta }$, $\delta_{\theta }^{2}$, $\sigma^{2}$, $v^{2}$, *R* are estimated. Aiming at the problem of unknown parameter estimation in the adaptive state space model based on UKF, the EM algorithm is used to estimate these parameters by using the voltage data from the initial time to the current time. The parameters are denoted by $\Theta =(\mu_{\theta },\;\delta_{\theta }^{2},\;\sigma^{2},\;v^{2},\;R)$. It should be noted that the essence of the EM algorithm is to approximate the maximum likelihood parameters estimation by maximizing the joint likelihood function under the premise of the posterior probability. The unknown parameters of the model can be estimated by referring to the method in reference [33].

## 4. Method of Degradation Prediction

### 4.1. Problem Description

The degradation of PEMFC is a long-term time accumulation process, so it is necessary to extract the long-term dependence of degradation data to predict the performance degradation trend of PEMFC. The transformer network solves the problem of long-term RNN dependency and can handle long sequences of inputs. In order to predict the degradation trend of PEMFC, we directly use the aging factor α filtered by UKF as a health index and predict it to evaluate the degradation degree.

The performance degradation prediction problem can be formally defined as follows. The input is $\alpha_{t}\in {ℜ}^{k},\;t=\left(1,\;2,\;\ldots ,\;T\right)$, where *T* is the length of α. The output is the label $Y_{t}$ for each time step prediction. The purpose is to establish a mapping relationship between input data α and label $Y_{t}$ to predict a real-time label, which can be expressed as

(8) $$Y_{t}=f\left(\alpha_{t}\right)$$

where *f* represents the mapping function, and $Y_{t}$ represents the real-time label predicted by *f*. In this paper, we use the transformer structure to establish the mapping.

### 4.2. The Transformer Structure

**Figure 1:**
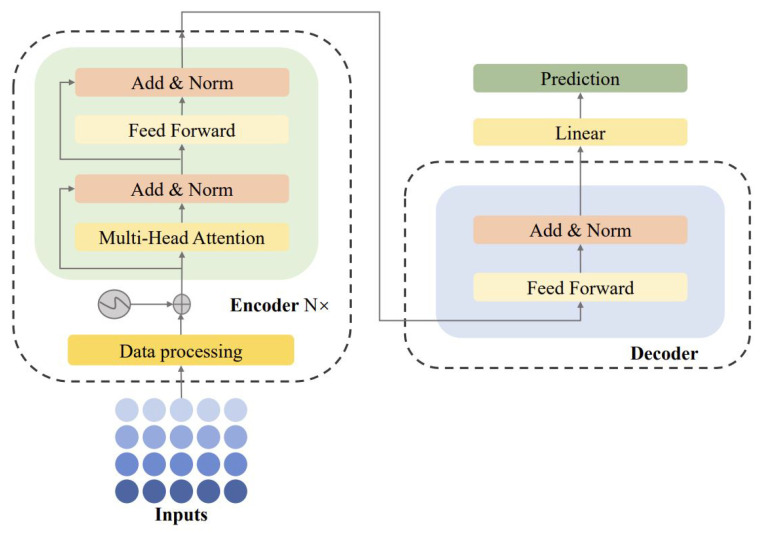
The structure of transformer network.

The network structure of transformer is composed of the attention mechanism. Instead of the sequential input of RNN, transformer feeds all of a sequence of data into the network at once for calculation, which brings good parallelism to the network model. Therefore, considering this advantage of transformer, we try to realize the performance degradation prediction based on the transformer network. The network structure is shown in Figure 1. The network consists of input, encoder, decoder and output.

#### 4.2.1. Data Input

Since transformer abandons the RNN structure and cannot adequately capture the sequential information of the feature sequences, position encoding is added before input. Therefore, the relative position information is added to the feature sequence by the position encoding technique and then is sent to an encoder to learn how the data points relate to each other and how important each time step is, resulting in more global characteristics. This paper adopts the most commonly used position encoding methods in transformers, namely sine position encoding and cosine position encoding [25]:

(9) $$\left\{\begin{array}{c} P\left(t,2i\right)=sin\frac{t}{10,000^{\frac{2i}{d}}},\\ P\left(t,2i+1\right)=cos\frac{t}{10,000^{\frac{2i}{d}}} \end{array}\right.$$

#### 4.2.2. Encoder

The encoder module consists of *N* encoders. Each encoder consists of three parts, including multi-head attention mechanism, feedforward neural network, residual connection and normalization layer.

The encoder is responsible for projecting the input feature sequence into a vector space that contains information about the input features through an embedding layer. The core of encoder is the self-attention mechanism. The self-attention mechanism learns the correlation between each sample in the input sequence and all other samples in the sequence, and calculates the similarity between vectors to capture internal dependencies, thus solving the problem of capturing long-term dependencies. The purpose of the self-attention mechanism is to screen out important information from the input feature sequence and assign greater weight to indicate the importance of capturing information, allowing the model to focus on more important information and improve the quality of output.

The self-attention mechanism uses the scaled dot product attention to calculate the attention value of the feature matrix. The query matrix and the key matrix are dotted with scaling and softmax normalization to calculate the weight of each value matrix, and the output attention is a weighted summation of the value matrix:

(10) $$Attention\left(Q,\;K,\;V\right)=soft\left(\frac{{\mathrm{QK}}^{T}}{\sqrt{d}}\right)V$$

(11) $$\left\{\begin{array}{c} Q=\alpha_{t}W_{Q}\\ K=\alpha_{t}W_{K}\\ V=\alpha_{t}W_{V} \end{array}\right.$$

where the Q is query matrix, K is the key matrix, and V is the value matrix. They are obtained by multiplying the input characteristic matrix $\alpha_{t}$ with the corresponding weight matrix $W_{Q}$, $W_{K}$ and $W_{V}$ respectively, and *d* is the dimension of Q, K and V.

**Figure 2:**
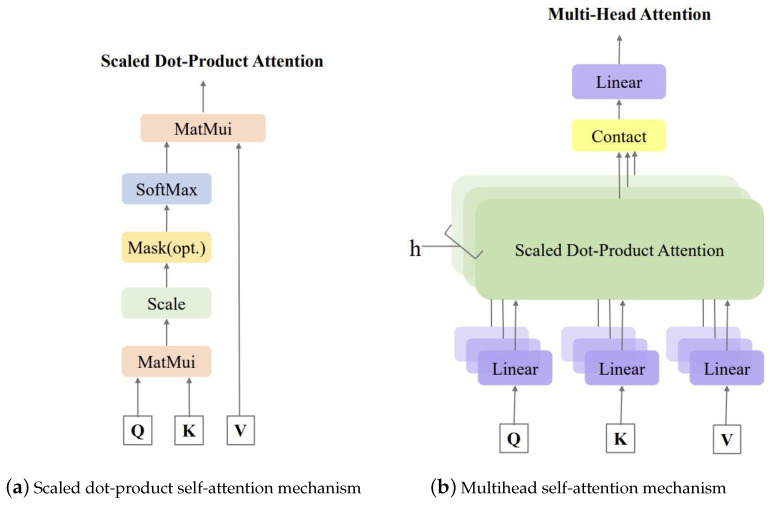
Process of multihead self-attention.

The self-attention mechanism can enable the network model to focus more on important feature information in the input sequence, while a single attention mechanism can only learn related information in one subspace. The multi-head self-attention mechanism can learn and focus on information from multiple subspaces, thereby obtaining richer features and information contained in the input sequence. The multi-head self-attention mechanism consists of several parallel and independent self-attention mechanisms. By concatenating and linearly transforming multiple attention values, the final attention value can be obtained, achieving multi-level and multi-perspective capture of the input sequence features. Figure 2 visualizes multihead self-attention, which can be expressed as

(12) $$MultiHead\left(Q,\;K,\;V\right)=concat\left(head_{1},\;head_{2},\ldots ,\;head_{h}\right)W$$

(13) $$head_{i}=Attention\left(\alpha_{t}W_{i}^{Q},\;\alpha_{t}W_{i}^{K},\;\alpha_{t}W_{i}^{V}\right)$$

where $W_{i}^{Q}$, $W_{i}^{K}$, $W_{i}^{V}$ are the weight matrix of the *i*th self-attention head, *h* is the number of heads of self-attention, and the contact function is used to concatenate the output value of each head’s attention calculated.

The feedforward neural network reduces the effect of long time sequence on the fitting degree of attention mechanism. The addition of residual structure can be used to prevent network degradation. Adding layerNorm can make the input of neurons in each layer have the same mean and variance, accelerate convergence and improve running speed.

#### 4.2.3. Decoder

The decoder is responsible for mapping the intermediate vector output by the encoder into an output sequence. The decoder module also consists of *N* decoders, each of which should include the mask multi-head self-attention mechanism, multi-head attention mechanism, feedforward neural network, residual connection and normalization layer. Unlike machine translation tasks, time series prediction does not require a layer of masked multiple attention, so the decoder layer is changed to a fully connected layer.

#### 4.2.4. Output

Since the input is numerical data, there is no need for a softmax function. After, the decoder directly outputs the prediction result.

### 4.3. The Method of MC-Dropout

The method based on deep learning can only provide the point estimation of degradation prediction as the prediction result, and cannot provide the confidence interval. The neural network will give a good prediction result in most cases after training, but will occasionally give a poor result. This kind of point estimate cannot quantify the deviation between the predicted value and the real value, which is of little significance to the application of PEMFC in practical engineering and has limited support for maintenance decisions. Therefore, it is not enough to calculate only the point estimation results of the degradation prediction, so it is necessary to obtain the probability interval of the prediction results.

There are two uncertainties in deep learning algorithms, cognitive uncertainty and accidental uncertainty. Cognitive uncertainty refers to the inability to obtain accurate models due to limited observations in practical applications. Accidental uncertainty usually refers to the uncertainty of the sensor measurement. With the consideration of these uncertainty sources, MC-dropout was proposed by Gal et al. [27]. The method of MC-dropout means that if the dropout is turned on during the test, the uncertain prediction results can be obtained by using the method of multiple Monte Carlo sampling. The number of neurons will be randomly discarded or adjusted according to the weight parameter, and the prediction results of each time will be different, so the probability distribution of the predicted results can be obtained.

Specifically, the Bayesian neural network is approximated by the dropout, and the probability distribution is approximated by Monte Carlo sampling to establish the uncertainty of prediction. During the training process, the data of α are selected to train the transformer network, and the dropout is turned on to effectively avoid the occurrence of the over-fitting phenomenon. Turn on the dropout during the prediction process, and the neurons in the hidden layer are temporarily dropped from the network according to a certain probability, making the predicted results different each time. A number of different point prediction results can be obtained by Monte Carlo sampling. The neural network model trained with dropout is iterated *n* times to obtain the mean and standard error of the predicted results *n* times. By utilizing the mean and variance, an approximation of the probability distribution for the uncertainty in degradation prediction can be obtained.

### 4.4. The Hybrid Prediction Method for Performance Degradation

**Figure 3:**
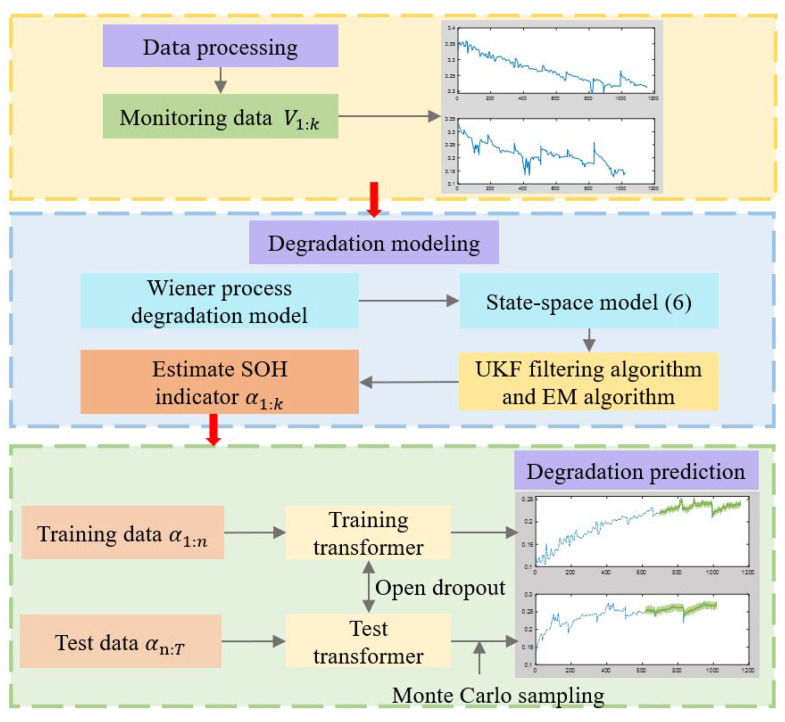
The hybrid method for degradation of PEMFC.

The hybrid method for the performance degradation prediction of PEMFC are shown in Figure 3. It includes three parts: data acquisition, degradation modeling based on the model-based method and degradation prediction based on the data-driven method. Firstly, the data monitored by the sensor are processed to obtain the voltage data. Then, a Wiener process model for aging factor α is established to characterize the degradation of PEMFC. The degradation state of PEMFC is estimated by the UKF algorithm and EM algorithm, and the estimation results of the unknown parameters of the model are obtained. Finally, the aging factor α obtained after filtering is divided into the training set and test set, and the degradation trend of PEMFC is predicted in the long term through the transformer network.

On the premise of knowing the voltage failure threshold of PEMFC, the failure threshold of the aging factor corresponding to the failure time can be determined, and the current health state of the PEMFC can be evaluated based on the prediction results of the neural network. The RUL also can be estimated by evaluating the time until the aging factor reaches the failure threshold. According to the estimated RUL, the appropriate maintenance measures can be taken in advance to reduce operating and maintenance costs and extend the lifespan of the PEMFC. At the same time, the RUL prediction results can serve as an important reference for system design and optimization.

## 5. Experimental Study

### 5.1. Experimental Dataset

The experimental datasets are obtained from the IEEE PHM Data Challenge 2014 provided by the French Federation for Fuel Cell Research FCLAB [34]. The data consist of two parts: FC1 and FC2, which are obtained under static and dynamic operating conditions, respectively. In order to ensure the stability of PEMFC operation, the flow rate of hydrogen and oxygen inlet and outlet are monitored in real time during testing, in order to avoid the instability of the output caused by too low or too high flow rates. The polarization curve test and electrochemical impedance spectroscopy (EIS) test of FC1 and FC2 are also conducted weekly to evaluate the performance status and ensure the stable operation of FC1 and FC2. More detailed information can be found in the description file of this dataset [34].

The proposed method is applied to the above experimental data, both the static dataset FC1 and the dynamic dataset FC2. The parameters of the transformer network are listed in Table 1.

### 5.2. State Estimation

**Figure 4:**
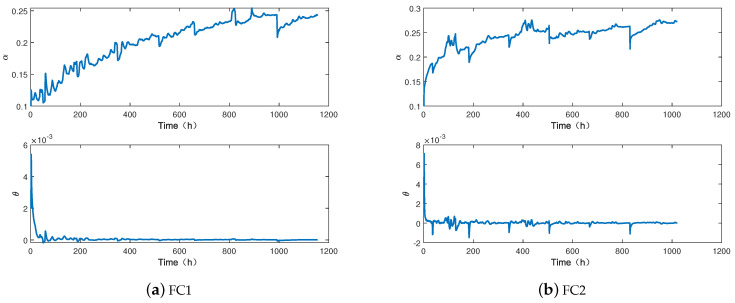
The results of state estimation.

The initial values of the state $X_{0}$, covariance matrix $P_{0}$, process error covariance matrix Φ, and measurement error covariance matrix *R* are set as follows: $X_{0}={\left[0.1,\;0.002\right]}^{T}$, $P_{0}=\left[10^{\left(-4\right)},\;0;\;0,\;10^{\left(-4\right)}\right]$, $\Phi =[10^{\left(-8\right)},\;0;\;0,\;10^{\left(-8\right)}]$, $R=10^{\left(-3\right)}$. The estimated results of aging factor α and drift rate θ of FC1 and FC2 are shown in the sub-figures in Figure 4a,b, respectively. The parameter α represents the degradation state, and a higher value means a more serious degradation degree of PEMFC. The parameter θ represents the degradation rate of PEMFC, and a larger value means a faster degradation rate. It can be seen that the aging factor α increases with time, and the drift rate θ fluctuates slightly.

**Figure 5:**
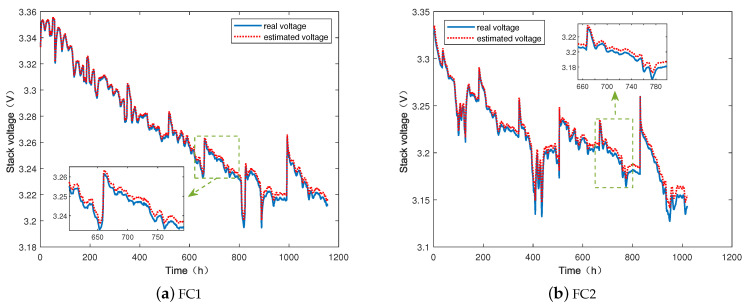
The fitting voltage curves.

Figure 5 shows the voltage fitting results after taking the aging factor α filtered by UKF into the semi-empirical aging model. The fitting voltage curves can accurately track the voltage degradation trend of PEMFC.

### 5.3. Performance Degradation Prediction Results

After estimating the aging state of PEMFC, the performance degradation prediction can be carried out by transformer with MC-dropout. To evaluate the efficacy of the MC-dropout approach, evaluation metrics, such as mean absolute percentage error (MAPE), root mean square error (RMSE), and mean absolute error (MAE) are used:

(14) $$MAPE=\frac{1}{k}\sum_{1}^{k}\frac{|Y_{t}-{\hat{Y}}_{t}|}{|Y_{t}|}$$

(15) $$RMSE=\sqrt{\frac{\sum_{1}^{k}{\left(Y_{t}-{\hat{Y}}_{t}\right)}^{2}}{k}}$$

(16) $$MAE=\frac{\sum_{1}^{k}\left|Y_{t}-{\hat{Y}}_{t}\right|}{k}$$

**Figure 6:**
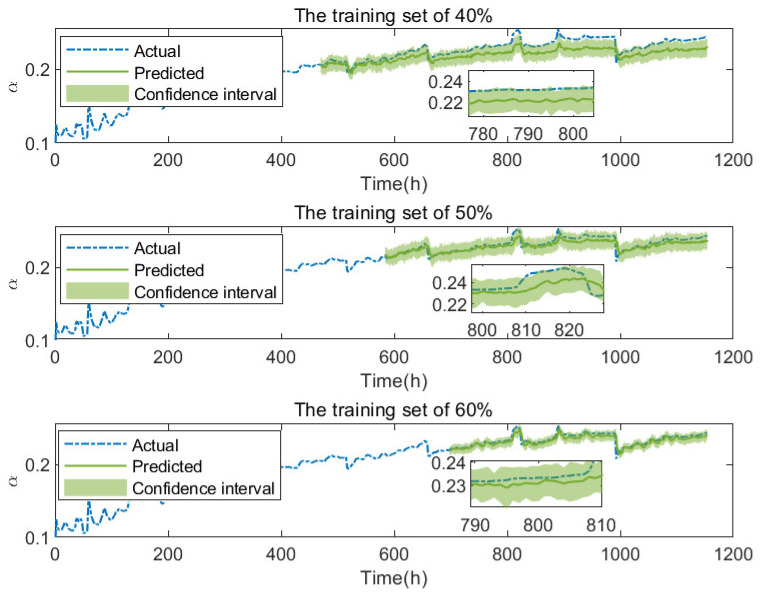
The degradation prediction of FC1 with different training set sizes.

**Figure 7:**
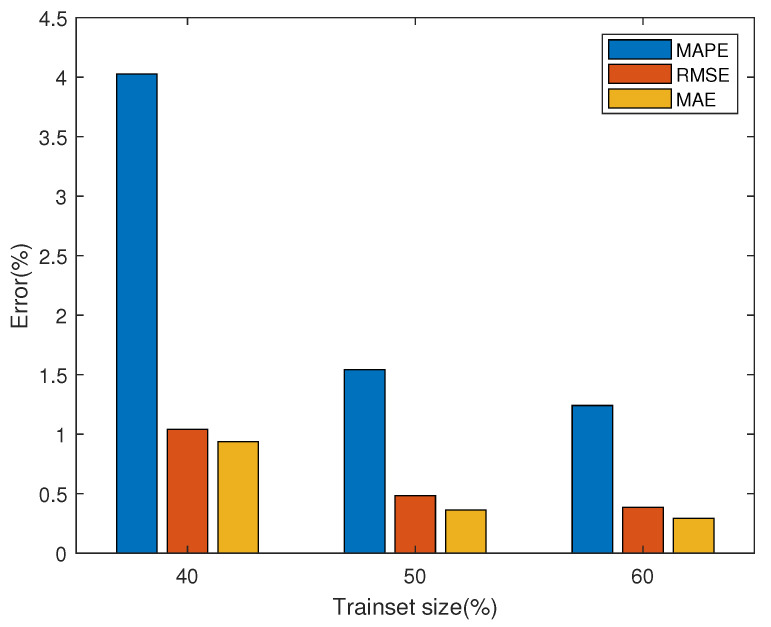
Error of FC1 for degradation prediction with different training set sizes.

Figure 6 shows the degradation prediction results of FC1 with different training set sizes (40%, 50%, and 60%). The output predicted value is the average of 100 iterations of the MC-dropout method in the optimal model after 50 epochs training. At the same time, the confidence interval made by the standard error obtained from MC-dropout is given. Figure 7 and Table 2 show the prediction performance of FC1 under different training set sizes. It can be seen from the figure that with the increase in training set data, the prediction performance gradually becomes better, and the three performance evaluation indexes MAPE, MAE and RMSE also decrease gradually. Since the prediction network can extract more information from more training data, it helps the subsequent network prediction task.

**Figure 8:**
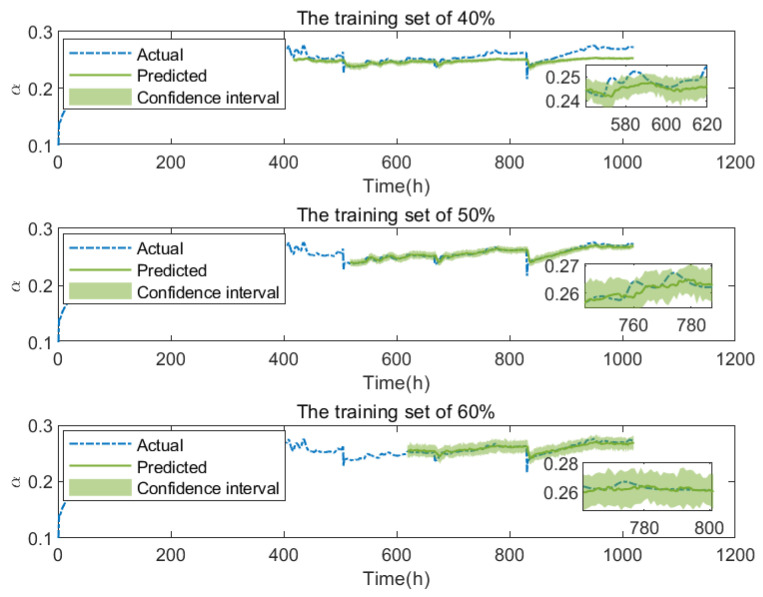
The degradation prediction of FC2 with different training set sizes.

**Figure 9:**
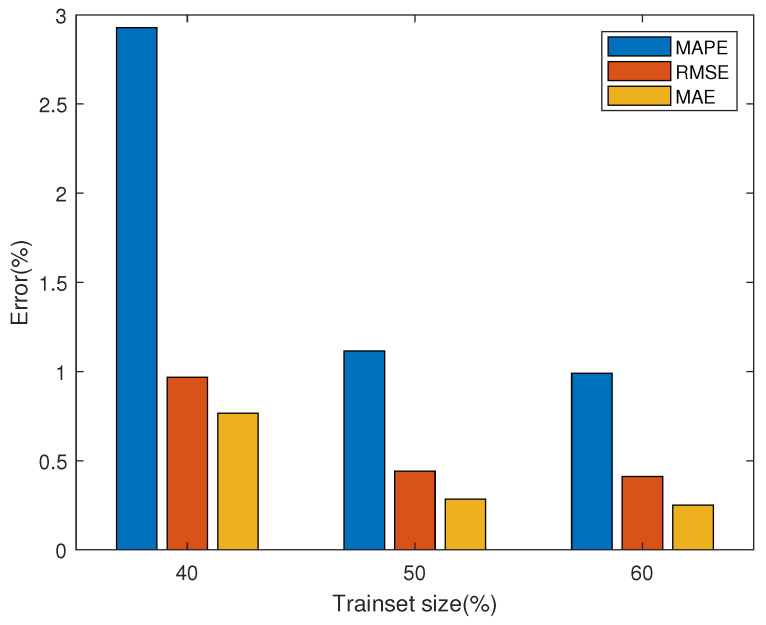
Error of FC2 for degradation prediction with different training set sizes.

The prediction results of FC2 under dynamic conditions are shown in Figure 8. Figure 9 and Table 3 give three evaluation indexes under different training set sizes. The predicted mean also fits the real value fairly well. It turns out that the average of all predictions provides a reliable estimate of the final prediction, whereas the standard error can serve as an indicator of the uncertainty of the model with respect to the input data.

### 5.4. Verification with LSTM

**Figure 10:**
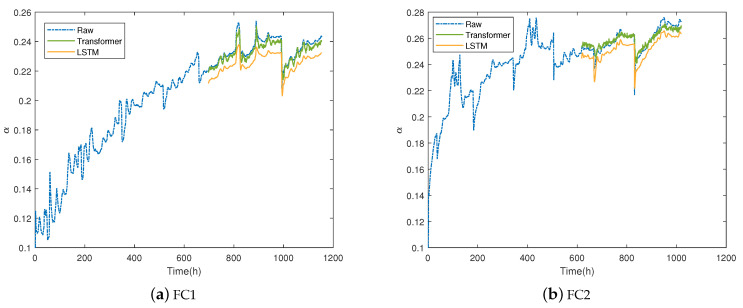
The result of PEMFC for degradation prediction with proposed method and LSTM.

The LSTM network is a classical method in time series data processing. In order to prove the superiority of the model proposed in this paper, LSTM model is also established to predict degradation trend. LSTM network uses two hidden layers, and the number of neurons in the first layer and the second layer is set to 100. The other parameters are the same as the transformer settings, and the training set size is 60%. The comparison results of FC1 and FC2 are shown in Figure 10. When 50 epochs are trained, it can be roughly estimated from the figure that the prediction error of LSTM is larger than that of transformer. When the transformer training is complete, the LSTM network structure has not been trained to its best. This is because transformer can process time series data in parallel and can quickly extract global information, whereas LSTM training is iterative.

## 6. Conclusions

In this work, a hybrid prediction framework is developed for the real-time performance degradation prediction of PEMFC, in which MC-dropout, combined with the transformer network, predicts the long-term degradation trend, and an adaptive Wiener process model with multi kinds of variability sources is established to model the degradation of PEMFC. The effectiveness and advantages of the framework are illustrated by experimental verification. While transformer network have advantages in the parallel processing of input data, the timing characteristics of time series are ignored when dealing with time series problems. Transformer only uses position encoding to determine the order of input data, whereas LSTM regards position information as the sequential input. Transformer does not fully achieve the effect of the sequential structure of LSTM in this respect. In the future, we will further consider combining these two network structures to improve the effectiveness of RUL prediction.

## Figures and Tables

**Table 1:** Parameters of transformer.

Parameters Setting	Value
Window length	10
Batch size	50
Epochs	50
Dropout	0.1
Multi-Head	10
Learning rate	0.001

**Table 2:** Prediction performance with different training set sizes for FC1.

Training Set	MAPE (%)	RMSE (%)	MAE (%)
40%	4.0261	1.0416	0.9380
50%	1.5429	0.4826	0.3630
60%	1.2410	0.3845	0.2923

**Table 3:** Prediction performance with different training set sizes for FC2.

Training Set	MAPE (%)	RMSE (%)	MAE (%)
40%	2.9276	0.9675	0.7665
50%	1.1153	0.4408	0.2851
60%	0.9896	0.4117	0.2515

## Data Availability

Not applicable.
